# Parasitic Infections as Chronic Drivers of Reduced Biological Efficiency in Sheep and Goats: Mechanisms, Consequences, and Mitigation Strategies

**DOI:** 10.3390/vetsci13101019

**Published:** 2026-09-26

**Authors:** Manyu Liu, Senyang Li, Lizhen Guo, Shucheng Huang, Fuchun Jian

**Affiliations:** 1 College of Veterinary Medicine, Henan Agricultural University, Zhengzhou 450046, China; manyuliu@stu.henau.edu.cn (M.L.); lsylee777@163.com (S.L.); 18211662116@163.com (L.G.); 2 Key Laboratory of Quality and Safety Control of Poultry Products, Ministry of Agriculture and Rural Affairs, Zhengzhou 450046, China; 3 Ministry of Education Key Laboratory for Animal Pathogens and Biosafety, Zhengzhou 450046, China

**Keywords:** sheep and goats, parasitic infections, host–parasite interactions, biological efficiency, pathophysiology, parasite control

## Abstract

Parasitic infections are major health and production challenges in sheep and goats. Their effects extend beyond acute clinical disease, as chronic and subclinical infections can cause persistent physiological disturbances that impair host function and reduce production performance. Although individual parasites and their associated diseases have been widely studied, how parasites with different life cycles, tissue tropisms, and pathogenic strategies ultimately converge on shared host responses remains less well integrated. This review examines major gastrointestinal, hepatobiliary and visceral, blood- and tissue-associated, and ectoparasitic infections of sheep and goats and the mechanisms linking parasitic infection to impaired host function. Distinct pathogenic processes include intestinal dysfunction, hematological impairment, organ and tissue dysfunction, nutritional inefficiency, and immune activation with metabolic trade-offs. Despite their different origins, these processes may be accompanied by overlapping downstream host responses, particularly oxidative stress, gut microbiota dysbiosis, and metabolic alterations, although the strength of evidence varies among parasite–host systems. Together, these changes may constrain biological efficiency—the capacity to acquire, utilize, and allocate resources toward physiological maintenance and productive functions—and thereby impair production performance. The consequences extend from individual animals to populations and production systems, including impaired growth and reproductive performance, reduced product quality, increased disease management costs, and less readily detected production losses that may accompany subclinical infection. Mitigation should remain grounded in established parasite-targeted control, complemented where appropriate by nutritional support and enhancement of host resistance or resilience. Microbiota-targeted interventions and technology-enabled precision management represent promising additional approaches, but their parasite-specific efficacy and practical applicability require further validation. By linking parasite-specific pathogenic processes with convergent host responses and production outcomes, this review provides an integrated framework for understanding parasite-associated losses and highlights the importance of combining parasite control with strategies that maintain or restore host function.

## Simple Summary

Parasitic infections are a major challenge to the health and productivity of sheep and goats worldwide. Their effects are not limited to severe disease or death. Long-lasting and mild infections that show few obvious signs can also reduce growth, reproductive performance, feed use, and the quality of meat, milk, and wool, leading to substantial but often overlooked production losses. This review summarizes the major parasitic infections affecting sheep and goats and explains how different parasites damage the intestine, blood, liver, muscles, skin, and other tissues. Although these parasites affect animals in different ways, the resulting changes can reduce the ability of animals to use nutrients and energy efficiently and ultimately decrease production performance. The review also discusses approaches to reduce these losses, including appropriate parasite control, nutritional support, breeding animals with greater resistance and resilience, management of beneficial gut microorganisms, and improved monitoring of animal health and parasite burden. Understanding parasitic infections from both animal-health and production perspectives can support more effective control strategies, improve animal welfare and productivity, and contribute to more sustainable sheep and goat production.

## 1. Introduction

Sheep and goat production plays a critical role in global livestock systems by providing meat, milk, wool, and breeding stock, particularly in marginal and semi-arid regions [1,2,3]. Beyond its nutritional contributions, it underpins rural economies and food security in many developing and pastoral regions [4,5]. Consequently, even marginal reductions in production efficiency translate into substantial economic losses at both farm and industry levels [6,7]. Parasitic infections are among the most pervasive health challenges in sheep and goat production systems worldwide [8,9,10]. The clinical expression and production consequences of parasitic infections vary substantially according to parasite species and pathogenicity, host susceptibility, infection intensity, nutritional and immune status, and environmental and management conditions. Although some infections can cause overt clinical disease and mortality, others may persist at subclinical levels under field conditions, with affected animals showing limited visible signs despite measurable physiological or production effects [11]. Consequently, production losses associated with subclinical infection may be difficult to detect using routine clinical assessment, although their magnitude and economic importance vary among parasite–host and production systems.

Current research has largely focused on epidemiology [9,12,13], parasite control strategies [14,15,16], and anthelmintic resistance in small ruminants [17,18,19,20]. These studies have advanced understanding of individual infections, including ovine coccidiosis and small-ruminant hemoparasitoses [21,22,23]. An integrative synthesis is nevertheless useful for relating parasite-specific pathology to host physiology and production efficiency. Consequently, a mechanistic synthesis remains lacking on how diverse parasitic infections collectively impair productivity via coordinated disruptions of intestinal integrity, hepatic function, hematological balance, immune regulation, metabolic homeostasis, and microbial ecology. This fragmented perspective hinders a comprehensive understanding of the persistent and cumulative production losses observed in sheep and goat populations under endemic parasitic pressure.

In this review, biological efficiency is operationally defined as the capacity of an animal to acquire, utilize, and allocate nutrients and metabolic resources to maintain physiological homeostasis while supporting productive functions, including growth, reproduction, lactation, and tissue deposition. This concept is broader than feed efficiency, which primarily relates productive output to feed input, and differs from production efficiency, which describes input–output relationships at the animal or production-system level. It is also distinct from performance or productivity, which represent observed productive outputs, and from resilience, which describes the ability to maintain or recover function and performance during or after a biological challenge. Biological efficiency is therefore not treated here as a single directly measurable trait, but as an integrative construct that can be evaluated through complementary indicators of nutrient utilization (e.g., feed intake, digestibility, and feed conversion), physiological maintenance and resource allocation, and productive outcomes (e.g., growth, reproductive performance, and product yield). In the context of parasitic infection, reduced biological efficiency may arise from impaired nutrient acquisition and utilization, increased maintenance and tissue-repair requirements, and redistribution of resources toward host defense, collectively reducing the resources available for productive functions.

This review integrates current knowledge on major parasitic infections affecting sheep and goat production systems, encompassing gastrointestinal, hepatobiliary and visceral, blood and tissue, and ectoparasitic groups, and examines their collective impacts on host physiological function and production efficiency. We develop a hierarchical framework to interpret parasite-associated economic losses, spanning individual-level declines in growth and reproductive performance, population-level instability driven by increased mortality and culling, and system-level hidden inefficiencies associated with subclinical reductions in feed utilization and management burdens. Building on this production-oriented framework, the review synthesizes the multi-organ and multi-system biological mechanisms underlying reductions in host biological efficiency. *Eimeria* spp. and *Haemonchus contortus* are used as mechanistic exemplars of intestinal epithelial injury and blood-feeding-associated hematological impairment, respectively. Other mechanisms considered include organ dysfunction induced by tissue-associated parasites, impaired nutrient utilization associated with gastrointestinal helminths, and immune–metabolic trade-offs associated with systemic parasitic infections. Potentially overlapping downstream host responses include oxidative stress, gut microbiota dysbiosis, and metabolic alterations, although the strength of evidence varies among parasite–host systems. Finally, mitigation strategies are considered according to their evidence maturity and practical applicability, distinguishing established parasite-targeted control from evidence-supported complementary nutritional and host-resilience approaches and from emerging microbiota-targeted and precision livestock strategies that require further validation.

## 2. Literature Search Strategy and Review Approach

This review was conducted as a narrative and integrative review rather than a systematic review. The relevant literature was identified through searches of PubMed, Web of Science, and Google Scholar, with the literature search last updated in September 2026. No publication-year restriction was applied. Search terms were used individually and in combination and included terms related to the target hosts (“sheep”, “goat”, and “small ruminant”), major parasitic infections and parasite groups (“*Eimeria*”, “*Haemonchus*”, “gastrointestinal nematodes”, “*Fasciola*”, “*Echinococcus*”, “*Babesia*”, “*Theileria*”, and “ectoparasites”), host responses and mechanisms (“intestinal barrier”, “anemia”, “oxidative stress”, “inflammation”, “gut microbiota”, “metabolism”, and “nutrient utilization”), production consequences (“growth”, “feed efficiency”, “reproduction”, “meat”, “milk”, “wool”, “production loss”, and “economic loss”), and mitigation strategies (“parasite control”, “anthelmintic resistance”, “nutrition”, “host resistance”, “resilience”, “microbiota”, and “precision livestock management”).

Peer-reviewed studies directly involving sheep and goats were prioritized, particularly those reporting parasite-associated physiological, production, or economic outcomes. Recent original research, systematic reviews, meta-analyses, and relevant guidelines were preferentially considered, while earlier studies were retained when they provided foundational evidence or information not adequately addressed by more recent literature. Studies in other host species were considered only when directly relevant evidence in sheep or goats was limited and when they provided useful mechanistic context; such evidence was interpreted cautiously and was not considered directly representative of small ruminants. Publications were excluded when they were not directly relevant to the parasite–host–production framework, lacked sufficient information to support the statements for which they were considered, or duplicated evidence available from more directly relevant sources. The selected literature was synthesized narratively according to parasite group, production consequence, host mechanism, and mitigation strategy, with attention to differences in parasite species, host species, infection context, and the strength and directness of the available evidence.

## 3. Major Parasitic Infections in Sheep and Goats and the Integrated Impacts on Host Physiology and Production Efficiency

Parasitic infections in sheep and goats constitute a complex multi-system biological stress that affects host performance through coordinated disruption of gastrointestinal [20,24], hepatobiliary and other visceral [25], hematological [23,26] and integumentary systems [27]. Although diverse in taxonomy and life cycle, these parasites converge on a common functional outcome: reduced nutrient utilization efficiency, impaired metabolic homeostasis, and decreased production performance (Table 1) [21,28,29,30]. Subclinical infections can also be associated with production losses, although the frequency and magnitude of these effects vary among parasite–host systems [30,31].

### 3.1. Gastrointestinal Parasites

Gastrointestinal parasites, including protozoa (*Eimeria* spp.), nematodes (*Haemonchus contortus*, *Ostertagia* spp., *Trichostrongylus* spp.), and cestodes (*Moniezia* spp.), can impair production through damage to gastrointestinal tissues, blood or protein loss, and altered nutrient utilization [28,30,32,33]. *Eimeria* spp. infection damages intestinal epithelial cells, leading to villus atrophy, tight junction disruption, and reduced absorptive surface area, which collectively result in malabsorption of nutrients and water imbalance [21,22,50]. In contrast, gastrointestinal nematodes, particularly *Haemonchus contortus*, exert additional systemic effects via blood feeding and protein loss, resulting in anemia, hypoproteinemia, and chronic energy deficiency [32]. These pathological changes are closely associated with reduced feed intake, poor feed conversion efficiency, and growth retardation in lambs [51]. Cestode can acquire nutrients from the intestinal environment, but evidence linking this process to quantified production losses is less developed than that for gastrointestinal nematodes [34,52]. Collectively, gastrointestinal parasites can impair growth and productive performance through multiple mechanisms, including epithelial injury, blood and protein loss, impaired nutrient absorption, and altered gastrointestinal function. The more detailed discussion of *Eimeria* spp. and *H*. *contortus* in Section 5 reflects their use as mechanistic exemplars of epithelial injury and blood-feeding-associated hematological impairment, respectively, rather than an assumption that these parasites are uniformly more important than other gastrointestinal parasites across production systems.

### 3.2. Hepatobiliary and Visceral Parasites

Hepatobiliary and visceral parasites can impair production through distinct forms of organ injury. *Fasciola hepatica* primarily affects the liver parenchyma and biliary system, where infection can induce chronic hepatic inflammation, bile duct injury, and disturbances in hepatic metabolic function [35,36]. These alterations may contribute to reduced growth performance, poorer body condition, and decreased feed efficiency [36]. In contrast, the metacestode stage of *Echinococcus granulosus* is not restricted to the liver. Hydatid cysts may develop in the liver, lungs, and other visceral organs, where progressive cyst enlargement can compress functional tissue and contribute to organ condemnation and reduced slaughter value [37,38,53]. Thus, the production consequences of these infections arise through different organ-specific mechanisms: hepatobiliary injury in fasciolosis and space-occupying visceral lesions in cystic echinococcosis.

### 3.3. Blood and Tissue Parasites

Blood and tissue parasites, including *Babesia* spp., *Theileria* spp., *Toxoplasma gondii*, and *Sarcocystis* spp., exert profound systemic effects on host physiology [23,26,40,42]. Hemoprotozoan infections cause hemolysis, anemia, and immune dysregulation, severely compromising oxygen transport capacity and metabolic efficiency. These effects frequently result in reduced growth performance, increased mortality during outbreaks, and substantial productivity losses [11,23,26]. Importantly, *Toxoplasma gondii* infection targets the reproductive system and placenta, leading to embryonic death, abortion, stillbirth, and reduced lambing rates [39,42,54]. Consequently, reproductive efficiency is significantly compromised, making this group of parasites a major driver of breeding-related economic losses in sheep production systems [42]. Tissue-dwelling parasites such as *Sarcocystis* spp. further contribute to economic losses by damaging skeletal muscle fibers, leading to deterioration in meat quality, including reduced tenderness and altered carcass appearance [40,41].

### 3.4. Ectoparasites

Ectoparasites, including ticks, mites, lice, and myiasis-causing flies, primarily affect sheep and goats through external blood loss, skin damage, and persistent irritation and stress [27,43]. Tick infestations are associated with chronic blood loss, and ticks can transmit other pathogens, amplifying systemic disease burden [11,23,26]. Infestations with mites and lice induce severe pruritus, dermatitis, and integumentary damage, which negatively affect wool quality and overall animal welfare [27]. The effects of myiasis depend on the site of infestation. Oestrosis, a form of nasal myiasis, is documented in sheep and goats, but its prevalence should not be interpreted as a quantitative estimate of production loss [44]. Collectively, ectoparasites contribute to reduced weight gain, lower wool quality, increased disease susceptibility, and elevated management costs [27,43].

**Figure 1:**
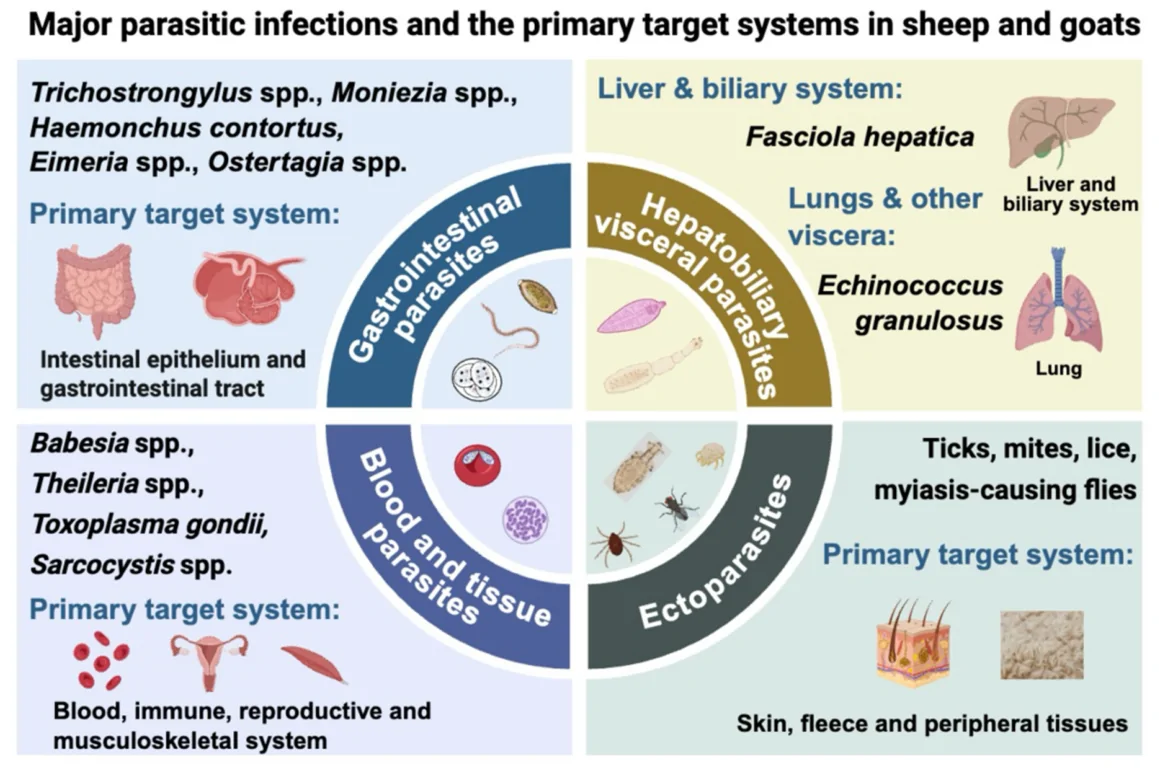
Major parasitic infections and their primary target systems in sheep and goats. Parasitic infections in sheep and goats encompass diverse groups, including gastrointestinal parasites (*Trichostrongylus* spp., *Moniezia* spp., *Haemonchus contortus*, *Eimeria* spp., and *Ostertagia* spp.), hepatobiliary and visceral parasites (*Fasciola hepatica* and *Echinococcus granulosus*), blood and tissue protozoa (*Babesia* spp., *Theileria* spp., *Toxoplasma gondii*, and *Sarcocystis* spp.), and ectoparasites (ticks, mites, lice, and myiasis-causing flies). These parasites exhibit distinct tissue tropisms. Hepatobiliary flukes primarily affect the liver and biliary system, whereas *E*. *granulosus* metacestodes can involve the liver, lungs, and other visceral organs.

Despite their biological diversity, parasitic infections can impair host function through distinct and context-dependent mechanisms, the nature and severity of which vary with parasite species, tissue tropism, infection intensity, host susceptibility, and environmental conditions. Depending on the parasite–host system, these effects may include impaired nutrient acquisition or utilization, hematological disturbance, tissue injury, and immune activation, while the broader mechanistic literature provides a conceptual basis for considering parasite-associated immunometabolic alterations [55]. When sufficiently severe or persistent, these physiological constraints can contribute to poorer feed efficiency, growth, reproductive performance, or product yield and quality [30,42,43]. However, the magnitude and economic significance of these outcomes vary considerably among infections and production settings, and direct quantitative evidence is substantially stronger for some parasite–host combinations than for others (Figure 1).

## 4. Hierarchical Production Consequences of Parasitic Infections in Sheep and Goats

Parasitic infections in sheep and goat production systems can generate production and economic losses that extend beyond overt clinical disease and mortality [7,30,56]. At the individual-animal level, these losses may reflect reductions in biological efficiency arising from impaired nutrient acquisition and utilization, increased maintenance demands, and altered resource allocation. Such host-level effects can subsequently manifest as reduced growth, reproductive output, and product yield or quality and, when sustained across animals and time, contribute to population- and system-level losses, including less readily detected losses associated with subclinical infection (Table 2). However, the magnitude and type of loss vary substantially according to the parasite–host combination, infection burden, production system, and environmental context, and not all proposed downstream consequences have been quantified economically. This hierarchical perspective distinguishes impairment of biological efficiency at the host level from its downstream production and economic consequences.

### 4.1. Individual-Level Losses

At the individual level, growth performance is among the most frequently evaluated production outcomes of parasitic infection [30]. The magnitude of the effect varies with parasite species and burden, host age and susceptibility, nutritional status, and production conditions. For gastrointestinal nematode infections in sheep, a systematic review and meta-analysis of 88 studies comprising 218 trials estimated that infected animals achieved approximately 85% of the weight gain, 90% of the wool production, and 78% of the milk yield of uninfected animals, although substantial variation among studies was evident [30]. These quantitative estimates provide relatively strong evidence for production effects of gastrointestinal nematodes, but they should not be extrapolated directly to other parasite groups or production systems. Parasite-specific evidence is also available for ovine coccidiosis; experimental and clinical studies indicate that pathogenic *Eimeria* infections can impair growth performance in susceptible lambs [21,33,60]. Mechanistically, gastrointestinal helminth infections can reduce productive performance through reduced feed intake, increased endogenous protein losses, and altered protein metabolism, thereby limiting nutrient availability for growth and other productive functions [61]. The relative contribution of these processes, however, varies among parasite–host systems and infection conditions.

Parasitic infections can impair reproductive performance through mechanisms that depend on the parasite and stage of reproduction [42]. In sheep, these include delayed puberty associated with gastrointestinal nematodes and pregnancy loss associated with placental protozoan infections [42,54]. A field study of subclinical gastrointestinal nematode infection found that benefits of pre-mating treatment varied with ewe body condition, supporting a context-dependent relationship between parasitism and fertility [57]. These findings should not be interpreted as evidence that all parasitic infections cause the same endocrine or reproductive disturbances.

Parasitic infections can affect product yield and, in selected systems, product quality. A meta-analysis documented reduced wool production and milk yield in sheep with gastrointestinal nematodes [30], and breed-dependent effects on milk productivity have been studied in dairy goats [59]. Evidence for meat-quality changes includes altered flavor-associated metabolites in lambs with *Sarcocystis* infection [41] and an experimental study of *Eimeria ovinoidalis* and aflatoxin B1 co-exposure [58]. The latter findings require interpretation within the co-exposure model and should not be attributed uniformly to parasitic infection alone. Ectoparasites also cause skin lesions in small ruminants and can reduce the value of animal products [27,43].

### 4.2. Population-Level Instability

At the population level, severe parasitic diseases can contribute to mortality or culling in susceptible flocks, particularly under conditions of high parasite burden, inadequate nutrition, or concurrent disease [11,32]. These effects are most relevant to highly pathogenic infections or vulnerable animal groups and should not be assumed to occur uniformly across parasite–host systems. Haemonchosis provides a representative example in sheep and goats, in which severe blood loss and anemia can result in substantial production and economic consequences [32,56]. Where mortality or culling occurs, the resulting changes in flock structure may increase replacement requirements and reduce long-term productivity. However, the magnitude of these population-level effects is highly context dependent, and robust quantitative estimates are unavailable for many parasitic infections and production systems.

### 4.3. System-Level Hidden Losses

Subclinical parasitic infections can generate production losses that are less readily detected than losses associated with overt disease [30,31]. Depending on the parasite–host system, reported effects include reduced feed efficiency or growth, prolonged production cycles, and other persistent production deficits that may not be apparent from clinical observation alone. Such effects are often described as “hidden” production losses; however, their economic magnitude has been quantified for only a subset of parasitic infections and production systems. Accordingly, these consequences should not be assumed to represent a uniform or dominant component of total parasite-associated economic burden across all settings.

More directly documented economic consequences include losses associated with reduced productive performance, organ condemnation, parasite-control measures, and ineffective treatment resulting from anthelmintic resistance. For example, an abattoir-based study of 384 slaughtered ruminants in Pakistan estimated annual losses of approximately PKR 133.7 million (USD 466,939) from condemnation of parasite-affected organs; however, the study included cattle and buffalo as well as sheep and goats, and the estimate therefore represents a context-specific ruminant-level rather than a sheep- or goat-specific economic burden [62]. Anthelmintic resistance can also impose measurable production costs in sheep: controlled field studies in lambs have demonstrated reductions in productive performance when animals were treated with an anthelmintic against which gastrointestinal nematodes were resistant [56], consistent with the broader production implications of anthelmintic resistance described for sheep gastrointestinal nematodes [63]. Thus, the economic consequences considered in this review include both directly measured production effects or costs and broader downstream consequences inferred from physiological or performance changes; the latter should be interpreted cautiously when direct economic quantification is unavailable.

**Figure 2:**
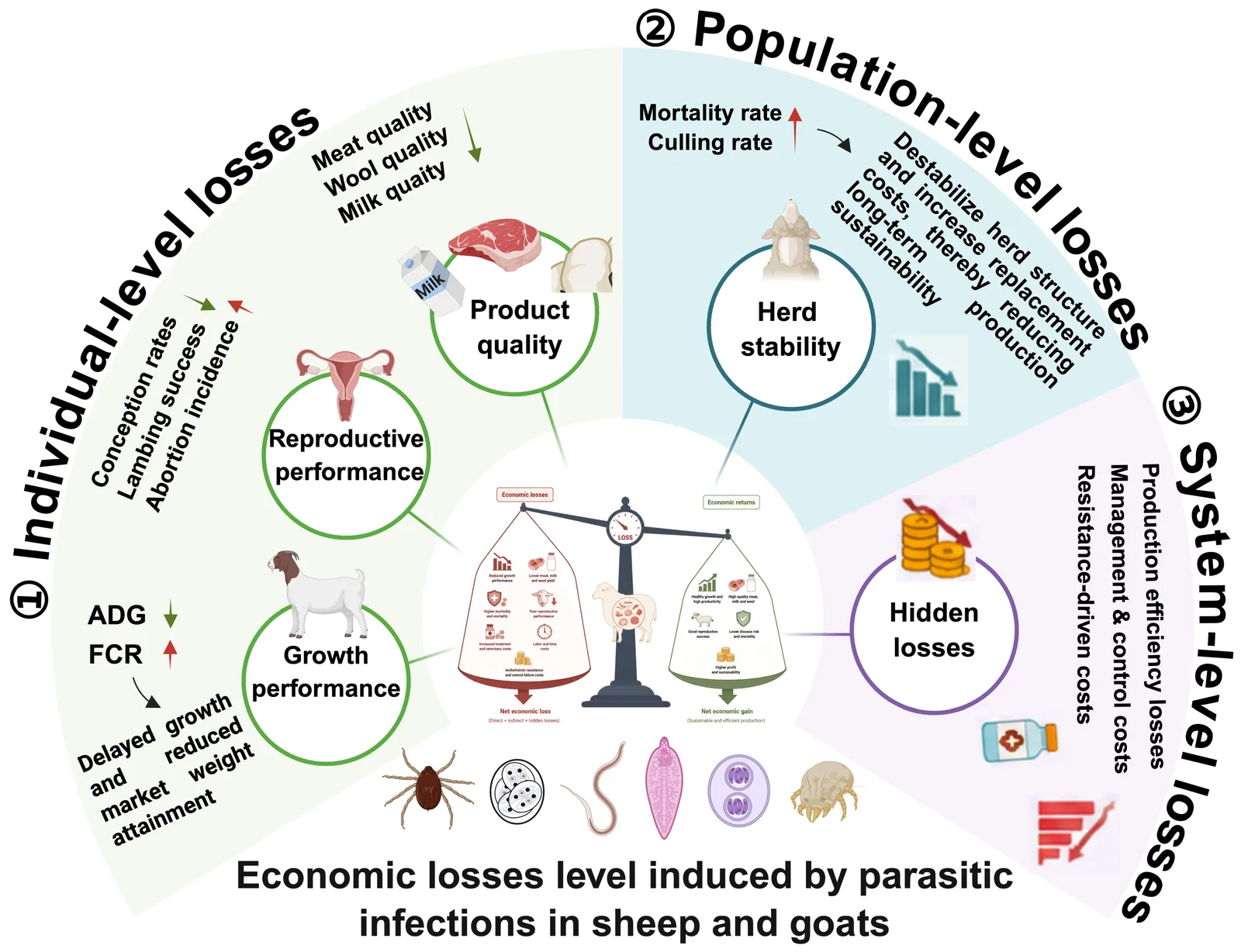
Hierarchical framework of documented and potential production and economic consequences of parasitic infections in sheep and goats. Parasite-associated effects may occur at individual, population, and production-system levels, although their occurrence and magnitude vary according to parasite species, host susceptibility, infection intensity, and production context. At the individual level, documented effects in specific parasite–host systems include reductions in growth, feed efficiency, reproductive performance, and product yield or quality. Severe or persistent infections may also contribute to mortality, culling, and replacement requirements at the population level. System-level consequences can include directly incurred control and management costs as well as less readily quantified production losses associated with subclinical infection. Solid evidence is available for selected parasite–host and production outcomes, whereas some broader economic consequences remain context dependent or incompletely quantified. Red upward arrows indicate increases, whereas green downward arrows indicate decreases in the adjacent variables. Black curved arrows indicate links between the labeled changes and their potential consequences. The pale-green, light-blue, and pale-purple sectors distinguish individual-, population-, and system-level losses, respectively. Outlined circles group the corresponding outcome domains. The central balance is a conceptual illustration of economic losses and returns, not a quantitative comparison. Animal, parasite, product, and monetary icons are schematic. ADG, average daily gain; FCR, feed conversion ratio. The illustrated changes are context dependent and should not be interpreted as occurring in every infected animal or production system.

Overall, available evidence indicates that parasitic infections can affect sheep and goat production at individual, population, and production-system levels, although the magnitude and relative importance of these effects vary markedly among parasite–host combinations and management contexts (Figure 2). Evidence is strongest where production traits or direct costs have been measured, whereas some broader population- and system-level consequences remain inferred from physiological or performance changes and have not been comprehensively quantified. Impaired host biological efficiency can contribute to these downstream production effects, but should not itself be equated with population- or system-level economic loss.

## 5. Biological Mechanisms Underlying Parasite-Induced Economic Losses in Sheep and Goat Production

The mechanistic sections below are organized around major modes of host impairment rather than around equivalent coverage of individual parasite taxa. Within this framework, *Eimeria* spp. and *Haemonchus contortus* are discussed in greater detail as mechanistic exemplars of intestinal epithelial injury and blood-feeding-associated hematological impairment, respectively, because these host–parasite systems are comparatively well characterized in small ruminants and provide clear links between parasite-specific pathology, physiological dysfunction, and production outcomes. Their use as exemplars is not intended to imply that they are representative of all parasitic infections or necessarily more important than other parasites across all epidemiological and production settings. Other parasite groups are incorporated where they illustrate distinct mechanisms, including organ and tissue dysfunction, impaired nutrient utilization, and systemic host responses.

### 5.1. Intestinal Epithelial Destruction and Nutrient Malabsorption Induced by Coccidia

**Figure 3:**
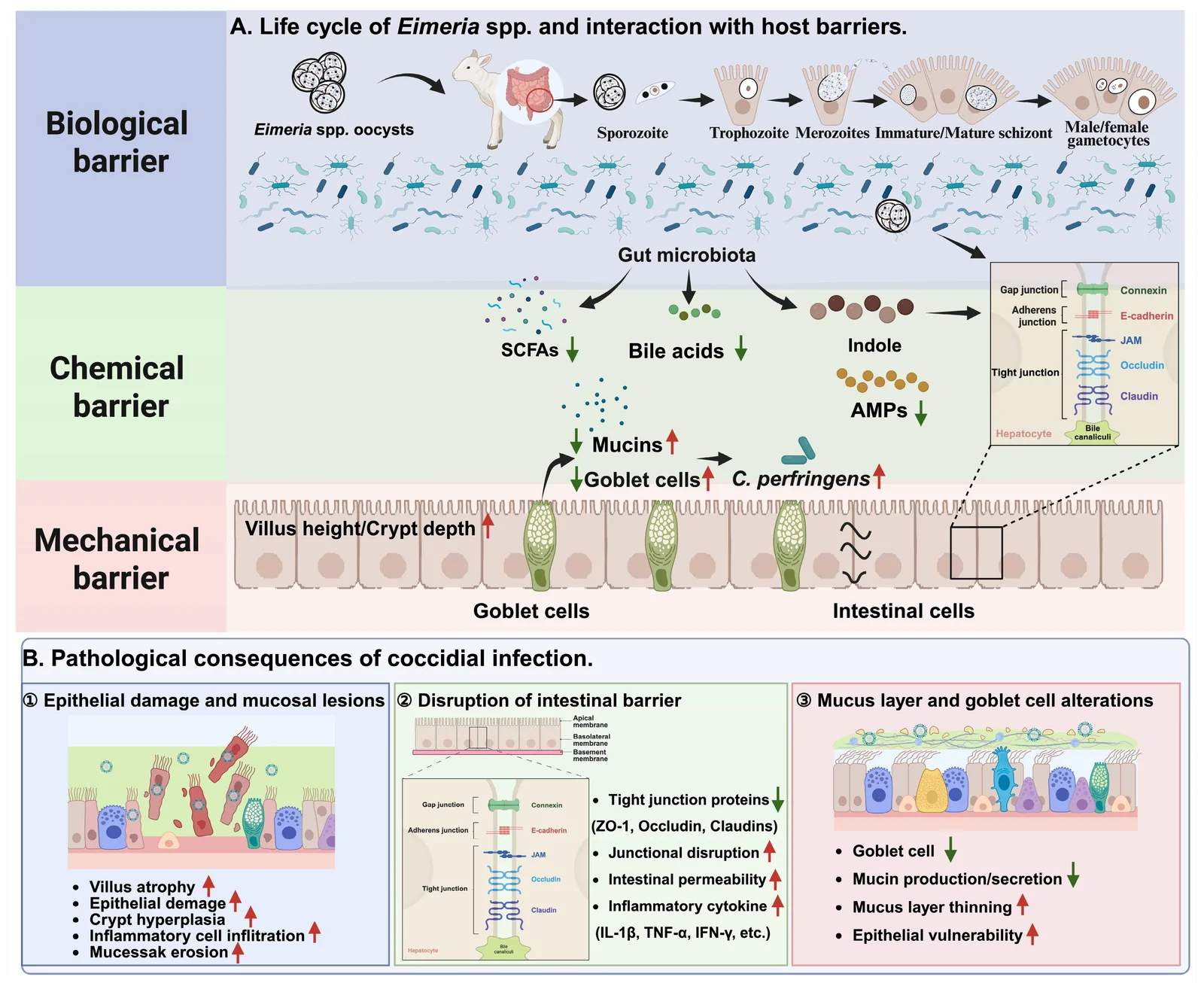
*Eimeria* spp. as a mechanistic exemplar of parasite-induced intestinal epithelial destruction and barrier dysfunction in sheep and goats. *Eimeria* spp. infection in sheep and goats disrupts intestinal epithelial integrity through intracellular parasite development, leading to villus atrophy, barrier dysfunction, mucus layer impairment, and increased intestinal permeability. These alterations reduce nutrient absorption and feed efficiency, ultimately contributing to impaired growth performance and cumulative production losses. In panel (**A**), the blue-purple, pale-green, and pink background bands distinguish the biological, chemical, and mechanical components of the intestinal barrier, respectively. Black arrows indicate the sequence of the illustrated parasite stages or proposed links among the labeled components. Red upward and green downward arrows indicate increases and decreases in the adjacent variables, respectively. Small colored circles in the chemical-barrier region schematically represent the metabolites or secreted molecules identified by the accompanying labels; their sizes and numbers do not indicate measured concentrations. The red circle on the animal marks the intestinal region illustrated, and dashed callout lines link an epithelial region to the enlarged junctional schematic. In panel (**B**), numbered boxes distinguish epithelial injury, barrier disruption, and mucus-layer or goblet-cell alterations. The depicted responses may vary with parasite species, infection stage, and experimental conditions. SCFAs, short-chain fatty acids; AMPs, antimicrobial peptides; ZO-1, zonula occludens-1; JAM, junctional adhesion molecule; IL-1β, interleukin-1 beta; TNF-α, tumor necrosis factor alpha; IFN-γ, interferon gamma.

Among the parasitic diseases affecting small ruminants, coccidiosis caused by *Eimeria* spp. is one of the most prevalent enteric infections in lambs and kids [9,13,33]. Economic losses associated with coccidiosis are often attributed to reduced growth performance, poor feed efficiency, and increased management costs [33,60]. Unlike many systemic parasitic infections, the primary pathological effects of coccidia originate within the intestinal epithelium, making intestinal damage a key link between infection and production loss [50] (Figure 3). Accordingly, *Eimeria* spp. are used here as a mechanistic exemplar to illustrate how parasite-induced intestinal epithelial and barrier damage can impair nutrient utilization and productive performance in small ruminants.

The pathogenic process begins when infective sporulated oocysts are ingested by the host [33,60]. Following excystation, sporozoites invade intestinal epithelial cells and undergo repeated rounds of intracellular development [33]. During schizogony and gametogony, parasite replication progressively destroys infected epithelial cells, ultimately leading to cell rupture and release of new developmental stages. The cumulative destruction disrupts normal mucosal architecture and reduces the functional absorptive surface [21,50,64]. Selected experimental challenge studies of pathogenic ovine *Eimeria* spp. have documented epithelial loss, villous and crypt lesions, and alterations in intestinal immune structures; however, the specific lesion patterns vary according to *Eimeria* species and strain, challenge dose, host age, sampling time, and experimental conditions [20,65,66]. Evidence from naturally occurring disease is more limited, although characteristic small-intestinal lesions have also been described in a field outbreak associated with *E*. *granulosa* [67]. Table 3 therefore summarizes selected, study-specific observations and explicitly distinguishes experimental challenge models from naturally occurring infection. These findings should not be interpreted as universally representative of coccidiosis in sheep and goats.

Beyond direct epithelial destruction, increasing evidence indicates that coccidial infection impairs intestinal barrier integrity [22,50] (Table 4). As general physiological background, the intestinal barrier is maintained by epithelial cells, mucus layers, and intercellular junctional complexes that regulate the movement of nutrients, water, and luminal antigens [68,69]. Experimental ovine studies have reported intestinal injury and changes in tight-junction-associated markers during *Eimeria* infection; results depend on challenge conditions, and aflatoxin co-exposure must be considered where present [21,22] (Table 3). Disruption of these structures compromises epithelial cohesion and increases intestinal permeability [70]. Concurrent inflammatory responses further exacerbate barrier dysfunction through the production of cytokines and other inflammatory mediators that alter epithelial turnover and junctional stability [21,22]. As a result, the intestine becomes less effective at maintaining selective permeability and mucosal homeostasis.

The mucus layer constitutes another important component that may be affected during coccidial infection [22,50]. Goblet cells and mucin secretion contribute to the physical separation of luminal contents from epithelial surfaces [68,78]. In lambs, goblet-cell and barrier alterations have been reported in an *Eimeria ovinoidalis*–aflatoxin B1 co-exposure model, with aflatoxin aggravating several infection-associated changes [22]. These observations support a possible contribution of mucus-barrier injury to intestinal dysfunction, but its magnitude and direction should not be generalized across all coccidial infections. Epithelial damage and inflammation provide plausible links to impaired nutrient utilization; direct quantification of individual nutrient absorption deficits in infected sheep and goats remains limited [21,33,50].

From a production perspective, intestinal injury provides a plausible link between coccidial infection and impaired performance. Ovine challenge studies document intestinal pathology and reduced growth, while reviews of small-ruminant coccidiosis describe diarrhea, dehydration, and production consequences in susceptible animals [21,33,60]. Reduced growth can prolong finishing and increase production costs, but these downstream costs should be distinguished from outcomes directly measured in individual studies. Coccidiosis therefore serves here as a mechanistic example linking epithelial and barrier injury to impaired host function and productive performance.

### 5.2. Blood-Feeding Parasitism and Anemia-Driven Reduction in Metabolic Capacity

Blood-feeding parasites represent an important cause of hematological impairment in small ruminants [24]. Among them, *Haemonchus contortus* is widely recognized as a highly pathogenic gastrointestinal nematode of sheep and goats [32] and is used here as a mechanistic exemplar of blood-feeding-associated blood loss and anemia. Unlike coccidia, which primarily damage the intestinal epithelium, *H*. *contortus* imposes a distinct physiological constraint through sustained blood loss, with consequent reductions in erythrocyte mass and oxygen-carrying capacity [32,45]. This well-characterized host–parasite interaction provides a useful model for examining how hematological impairment can constrain physiological function and productive performance [32,45].

The abomasal nematode *Haemonchus contortus* attaches to the mucosa and feeds directly on host blood throughout its parasitic stages. Although individual worms consume only small quantities, heavy infections can result in substantial cumulative blood loss [32] (Table 4). Persistent hemorrhage often leads to reductions in packed cell volume, hemoglobin concentration, and circulating erythrocyte numbers [32,45]. In young animals and under conditions of inadequate nutrition, these effects can progress to clinically significant anemia. Anemia has important implications for animal productivity because oxygen delivery is essential for normal cellular metabolism [32]. Reduced oxygen transport limits the metabolic activity of highly active tissues, including skeletal muscle and reproductive organs. As a result, infected animals frequently exhibit lethargy, reduced grazing activity, decreased feed intake, and slower growth rates. Blood loss is often accompanied by protein depletion [28,31]. Plasma proteins lost must be replaced through increased synthesis, creating an additional nutritional burden [28,32,71]. In growing lambs and kids, this process may compromise muscle deposition and weight gain [51,79]. In breeding animals, chronic anemia and protein deficiency can negatively affect reproductive performance by reducing body condition and limiting the resources available for reproductive processes [42,57].

The consequences extend beyond individual performance. Severe haemonchosis can cause substantial anemia and mortality in susceptible sheep [32]. In endemic production systems, repeated infections necessitate additional veterinary interventions and anthelmintic treatments, increasing management costs and labor [7,80]. Therefore, blood-feeding parasites can reduce production efficiency through chronic blood loss, anemia, and reduced oxygen delivery. The resulting decline in growth, reproduction, and survival contributes substantially to the economic burden.

### 5.3. Tissue Migration and Organ Dysfunction Leading to Long-Term Metabolic Impairment

Organ-associated parasites differ in their routes of establishment and pathological effects. *Fasciola hepatica* and *Fasciola gigantica* can cause injury during migration through hepatic tissue, whereas *Echinococcus granulosus* metacestodes form space-occupying visceral lesions [35,36,37]. *Dicrocoelium dendriticum* inhabits the biliary system without the parenchymal migration characteristic of *Fasciola*, and its clinical effects in sheep should not be assumed equivalent [36].

Liver flukes are among the most extensively studied tissue-migrating parasites in ruminants [35,36]. Following ingestion of infective metacercariae, juvenile flukes penetrate the intestinal wall and migrate through the liver parenchyma before establishing in the bile ducts [36,81]. This migration causes mechanical tissue damage, hemorrhage, and inflammatory responses [35] (Table 4). During chronic infection, persistent irritation of the biliary system promotes fibrosis, bile duct hyperplasia, and progressive deterioration of hepatic architecture [36]. The general physiological literature establishes roles for the liver in amino acid metabolism and the regulation of feed intake [82,83]. In sheep, chronic fluke infection is associated with hepatic injury, wasting, and poor body condition [36]. These findings support a link between organ damage and impaired performance, but the quantitative contribution of individual hepatic metabolic pathways to parasite-associated production losses remains incompletely resolved.

Hydatid cysts caused by *Echinococcus granulosus* represent another example of organ-associated production losses. Although many infections remain clinically unapparent, cyst development can occupy functional tissue space within the liver and lungs [37]. Progressive enlargement may impair organ function and reduce the value of edible organs at slaughter [38]. The effects are often chronic and cumulative. Unlike acute enteric infections that may produce obvious clinical signs, organ damage can develop gradually over extended periods. Consequently, reductions in productivity may remain unnoticed until substantial physiological impairment has occurred, contributing to the underestimation of losses from chronic parasitic infections.

From a production perspective, tissue-migrating parasites reduce efficiency primarily through organ dysfunction rather than direct nutrient competition or blood loss [37,38]. Damage to metabolically important organs such as the liver limits the animal’s ability to process nutrients efficiently, maintain normal physiological functions, and sustain productive performance [36,82,83]. Directly documented consequences in sheep and goats include condemnation of affected organs and associated financial losses [25,38]; broader metabolic and growth consequences depend on the infection and its severity.

### 5.4. Gastrointestinal Parasites and Impaired Host Nutrient Availability and Utilization

A number of gastrointestinal parasites reduce host productivity through mechanisms that extend beyond tissue destruction and blood loss. Among these, cestodes and several gastrointestinal nematodes can influence animal performance by competing for nutrients and by altering digestive efficiency [30,39,72] (Table 4). Although the direct nutritional demands of individual parasites are relatively small compared with those of the host, persistent parasitism may contribute to reduced nutrient availability and impaired feed utilization, particularly in young animals and under limited nutritional supply [71,72].

Cestodes such as *Moniezia expansa* acquire nutrients from the intestinal environment, and studies have examined their lipid accumulation and mineral concentrations relative to those of sheep hosts [34,52]. These findings establish biological interactions with host nutrient resources, but do not by themselves quantify reductions in host growth or feed efficiency. Nutrient competition should therefore be presented as a possible contribution whose production significance requires parasite- and burden-specific evidence.

Gastrointestinal nematodes, including *Trichostrongylus* spp., *Teladorsagia circumcincta*, and *Nematodirus* spp., further contribute to feed inefficiency through their effects on gastrointestinal physiology [71]. These parasites can alter digestive secretions, reduce digestive enzyme activity, and impair nutrient absorption by damaging the mucosal surface [73] (Table 4). Even when overt clinical signs are absent, infected animals often exhibit reduced feed conversion efficiency and lower weight gain compared with uninfected counterparts [30,73]. The consequences of parasitic infection are not limited to direct nutrient competition; host responses can also modify feeding behavior and digestive function [73,84]. Reduced appetite, altered rumen activity, and changes in gastrointestinal transit time have all been reported during parasitic infections [28,73,84]. Consequently, nutrient intake may decline while maintenance requirements increase, further reducing the proportion of dietary energy available for productive functions.

The combined effects are particularly important in growing lambs and kids, where rapid tissue accretion depends on efficient feed conversion. Persistent reductions can prolong the time to market weight and increase feed costs per unit of production. Therefore, parasites affecting nutrient availability and utilization can contribute to economic losses through persistent reductions in production efficiency, even in the absence of overt clinical disease. Overall, cestodes and gastrointestinal nematodes illustrate how relatively subtle alterations in nutrient availability and digestive efficiency can accumulate over time and reduce productivity.

### 5.5. Systemic Parasitism and Immune–Metabolic Trade-Offs in Resource Allocation

Systemic parasites exert their effects through mechanisms that differ substantially from those of enteric parasites. Blood-borne protozoa, including *Babesia* spp. and *Theileria* spp. do not primarily impair productivity through intestinal dysfunction or direct nutrient competition. Instead, they induce systemic physiological alterations that disrupt host metabolism, immune regulation, and resource allocation [47] (Table 4). As a result, production losses are often associated with the energetic costs of maintaining host defense and physiological homeostasis during infection.

*Babesia* and *Theileria* infections involve blood cells and, depending on the species, other cellular stages [23,26]. Studies of sheep naturally infected with *Babesia ovis* have documented changes in inflammatory markers, cytokines, and oxidative status [46,74]. General resource-allocation theory and the ruminant nutrition–parasite literature provide a framework in which immune defense and tissue repair compete with productive functions for nutrients and energy [71,75,85]. This framework is useful for interpreting possible immune–metabolic trade-offs, but it does not establish the magnitude of an energetic cost for every hemoparasite infection in sheep or goats.

Fever, anorexia, and anemia during small-ruminant hemoparasitic infections can impose physiological constraints and contribute to poor condition or impaired performance [11,23,26]. Evidence from sheep also links parasitic infection to reproductive outcomes, including context-dependent associations between gastrointestinal nematode control, ewe body condition, and fertility [42,57]. These sheep-specific findings should not be interpreted as direct proof of the same reproductive mechanism in every *Babesia* or *Theileria* infection. Continued allocation of resources to recovery is a plausible explanation for delayed restoration of performance, rather than a uniformly demonstrated post-infection outcome [75].

From an economic perspective, systemic parasitic infections illustrate how production losses can arise through altered resource allocation rather than direct tissue destruction alone. By increasing the metabolic costs of host defense and reducing the resources available for productive functions, systemic parasitism may contribute to lower biological efficiency and reduced economic returns. This mechanism provides an important example of how parasitic infections can influence livestock productivity through changes in host physiology and energy allocation.

### 5.6. Shared and Emerging Downstream Host Responses Associated with Parasite-Induced Production Losses

Despite substantial differences in parasite life cycle, tissue tropism, infection intensity, and pathogenic mechanisms, evidence from different parasite–host systems indicates that some downstream host responses may overlap. Among the most frequently reported are oxidative stress, alterations in gut microbial communities, and changes in host metabolism [21,22,45,46,47,48,49]. However, the evidence supporting these responses is neither uniform nor equally established across parasite species, host species, and infection contexts. Oxidative imbalance has been documented across several parasitic infections of sheep and goats, whereas evidence for microbiota disruption is concentrated largely in gastrointestinal infections and remains heterogeneous in the specific microbial changes reported. Evidence linking altered microbial metabolites, including short-chain fatty acids (SCFAs), or systemic metabolic reprogramming directly to parasite-associated production losses is comparatively limited and is derived predominantly from experimental or associative studies. Accordingly, these processes are considered here as overlapping downstream host responses with different levels of evidential support, rather than universal pathways shared by all parasitic infections.

#### 5.6.1. Oxidative Stress Across Parasitic Infections

Among the downstream responses considered here, oxidative imbalance is comparatively well documented across several parasitic infections of small ruminants, including coccidiosis, gastrointestinal nematode infections, fasciolosis, and blood-borne protozoan diseases [22,45,46,77,86]. Under normal physiological conditions, oxidants and antioxidants are maintained in a dynamic balance. However, excessive production of oxygen species (ROS) may overwhelm endogenous antioxidant systems, leading to oxidative damage of lipids, proteins, and nucleic acids during parasitic infection [87]. Studies of infected sheep and goats have reported altered antioxidant defenses and increased markers of oxidative damage, although the specific markers and their direction of change vary among studies [45,46,77]. Oxidative imbalance may extend beyond cellular injury and has been associated with impaired intestinal epithelial integrity, altered tissue metabolism, and hepatic dysfunction [50,88]. However, evidence directly linking parasite-induced oxidative stress to quantitative production losses remains less extensive than evidence documenting changes in oxidative status itself. Oxidative stress should therefore be regarded as a biologically plausible and relatively well-supported contributor to host dysfunction rather than as a universal or independently sufficient explanation for reduced animal performance.

#### 5.6.2. Gut Microbiota Dysbiosis and Disruption of Microbial Homeostasis

Increasing evidence indicates that parasitic infections can be accompanied by alterations in the composition and function of the gastrointestinal microbiota [21,22,47,50]. However, this evidence is strongest for gastrointestinal parasites, whereas comparable data for hepatic, blood-borne, tissue, and ectoparasitic infections in sheep and goats remain more limited. Moreover, the direction and magnitude of microbial changes vary among parasite species, host species, infection stages, diets, and experimental conditions [47,50,89]. Consequently, no uniform parasite-associated microbiota signature has yet been established across small-ruminant parasitic infections.

Altered microbial communities may nevertheless have functional relevance because the gut microbiota contributes to digestion, nutrient utilization, immune regulation, and maintenance of intestinal integrity [90]. Several studies have reported changes in fermentative or potentially beneficial taxa and enrichment of taxa associated with intestinal disturbance during parasitic infections [21,47,48,91,92]. However, most available studies are associative and cannot determine whether microbiota alterations are a cause of host dysfunction, a consequence of parasite-induced intestinal damage and altered nutrient flow, or both. Broader mechanistic reviews propose bidirectional parasite–microbiota interactions [89,93], but direct causal and production-level validation in sheep and goats remains limited.

Alterations in microbial metabolites, including short-chain fatty acids (SCFAs), are a possible functional consequence of microbiota disruption. The general mechanistic literature describes roles for acetate, propionate, and butyrate in epithelial metabolism and immune regulation [94]. Experimental volatile-fatty-acid administration in dairy goats also provides evidence of effects on epithelial inflammation and barrier function, but this was not a parasite-treatment study [95]. In sheep, *Eimeria* challenge and co-exposure studies have reported changes in microbial communities and metabolite profiles [21,22,49]. These observations do not establish a uniform reduction in SCFA production: changes in putative SCFA-producing taxa are not equivalent to direct metabolite measurements. Evidence that parasite-associated SCFA changes causally mediate impaired production in sheep and goats remains limited. This proposed mechanism therefore requires direct metabolite measurements and intervention studies.

#### 5.6.3. Metabolic Alterations and Resource Reallocation During Parasitic Infection

Metabolic adaptation is an expected component of the host response to infection because nutrients and energy are redistributed among competing demands, including immune defense, tissue repair, physiological maintenance, and productive functions [71,75,85,96]. Ovine studies have reported infection-associated differences in metabolite profiles, serum proteins, protein digestion, and amino acid allocation [21,48,49,76]. However, the direction and magnitude of these changes vary with parasite species, infection stage and intensity, host nutritional status, and experimental context. The current evidence therefore supports parasite-associated metabolic alteration, but does not establish a single conserved metabolic reprogramming pattern across parasitic infections of sheep and goats. Increased allocation of resources toward host defense and recovery provides a plausible framework for linking these metabolic changes to reduced productive performance [71], although direct causal evidence connecting specific metabolic pathways to production traits remains limited.

**Figure 4:**
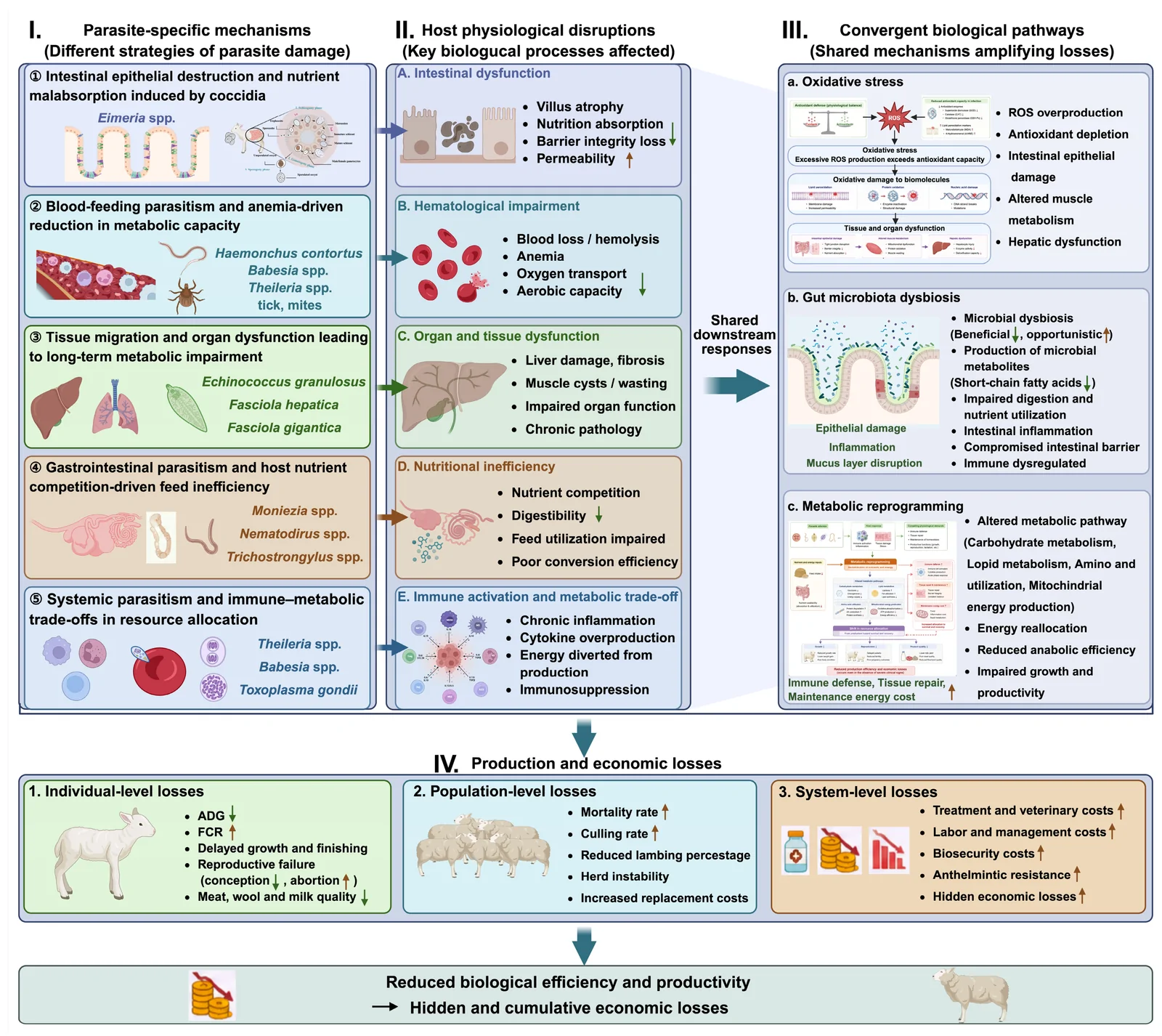
Multi-system biological mechanisms linking parasite infection to impaired biological efficiency and downstream production losses in sheep and goats. Parasite-specific pathogenic processes, including intestinal epithelial injury, blood loss, tissue invasion, impaired nutrient acquisition, and immune-metabolic disturbances, impose physiological and metabolic constraints on the host. These processes may be accompanied by overlapping downstream responses, including oxidative imbalance, gut microbiota dysbiosis, altered microbial metabolites, and host metabolic alterations. The strength and directness of evidence for these responses vary substantially among parasite species, host species, infection intensities, and experimental or field contexts; therefore, they should not be interpreted as universal or equally established mechanisms. Where present, these responses may constrain nutrient and energy utilization, increase maintenance requirements, and redirect resources toward host defense and tissue repair, thereby impairing host biological efficiency. The resulting host-level effects may subsequently contribute to poorer growth, reproductive performance, and product yield or quality and, when sustained across animals and time, to population- and system-level production losses. Panels (**I**–**IV**) organize the framework into parasite-specific mechanisms, host physiological disruptions, potentially overlapping downstream responses, and production and economic consequences, respectively. Matching colors in panels (**I**,**II**) link the corresponding mechanistic categories; the green, light-blue, and orange boxes in panel (**IV**) distinguish individual-, population-, and system-level consequences. Background colors are used for visual grouping and do not indicate effect size or evidence strength. Connecting arrows indicate proposed links within the conceptual framework rather than uniformly established causal relationships. Upward (↑) and downward (↓) arrows indicate increases and decreases in the labeled variables, respectively. Numbers and letters identify categories and subpanels. Icons schematically represent parasites, host tissues and cells, biological processes, livestock, and economic outcomes; their sizes and numbers are not quantitative. ADG, average daily gain; FCR, feed conversion ratio; ROS, reactive oxygen species.

Taken together, the current evidence supports a hierarchy rather than equivalence among these downstream responses. Oxidative imbalance is documented across several parasitic infections of small ruminants, whereas evidence for microbiota dysbiosis is stronger in gastrointestinal infections and remains heterogeneous across host–parasite systems. Altered SCFA metabolism and coordinated systemic metabolic reprogramming are mechanistically plausible but currently supported more strongly by associative and experimental evidence than by direct causal studies linking these changes to production outcomes. These responses may interact to constrain nutrient and energy utilization and alter resource allocation among physiological maintenance, host defense, tissue repair, and productive functions. Accordingly, they are best interpreted as overlapping and context-dependent pathways that may contribute to reduced host biological efficiency and subsequent production losses, rather than as universal mechanisms shared equally across all parasitic infections (Figure 4).

## 6. From Parasitic Burden to Efficiency Restoration in Sheep and Goat Production Systems

**Figure 5:**
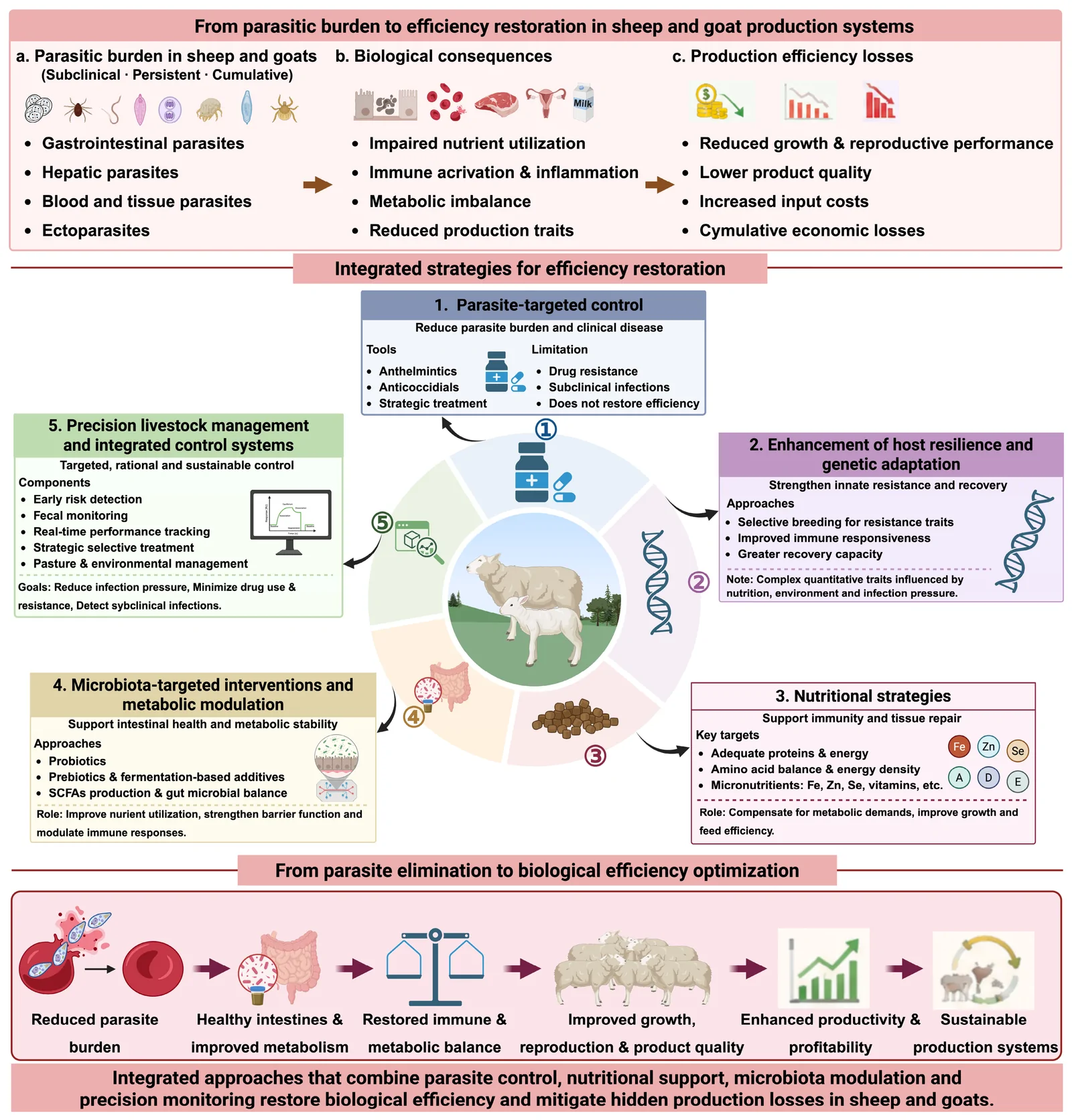
Evidence-informed framework for parasite management and support of host biological efficiency in sheep and goats. Intervention categories differ substantially in evidence maturity and readiness for routine implementation. Parasite-targeted and established integrated management measures constitute the core of current parasite control. Nutritional support and host genetic resistance or resilience represent evidence-supported complementary strategies whose applicability depends on production context. Microbiota-targeted interventions and technology-enabled precision livestock approaches are promising but remain less validated for parasite-specific outcomes and require further field evaluation before routine implementation. The framework therefore illustrates complementary roles across different levels of evidence rather than equivalent efficacy or practical readiness among all intervention categories.

Parasitic infections can constrain host biological efficiency by impairing nutrient acquisition and utilization, increasing physiological maintenance requirements, and altering the allocation of metabolic resources [28,71]. These host-level effects may subsequently be expressed as poorer growth, reproductive performance, and product yield or quality and, when persistent, contribute to reduced productivity at the production-system level [11,30,31]. Consequently, parasite management should consider not only reduction in parasite burden but also maintenance or recovery of host physiological function and productive capacity. However, the strategies discussed below differ substantially in the maturity of supporting evidence and their readiness for routine field application. Established parasite-targeted and integrated control measures remain the foundation of parasite management. Nutritional support and host genetic resistance or resilience represent evidence-supported complementary approaches, although their effectiveness is context dependent and they do not replace direct parasite control. By contrast, microbiota-targeted interventions and technology-enabled precision livestock approaches remain emerging strategies for which parasite-specific efficacy, scalability, and practical value require further field validation (Figure 5).

### 6.1. Parasite-Targeted Control and Current Limitations

Parasite-targeted control remains the established foundation of parasite management in small ruminants. Anthelmintic and anticoccidial efficacy must be considered separately, with drug choice informed by the parasite, host, and local treatment response [60,97]. Pharmacological treatment is incorporated into integrated programs that combine surveillance, targeted treatment, and pasture or environmental management [98,99,100]. Systematic reviews and meta-analyses document anthelmintic resistance in gastrointestinal nematodes of sheep and goats, with substantial regional and drug-class variation [17,18,20]. Toltrazuril resistance has also been confirmed in a field isolate of ovine *Eimeria*, but this observation should not be generalized to all coccidia or production systems [101]. The 2023 WAAVP guideline provides standardized methods for assessing anthelmintic resistance using the fecal egg count reduction test, while the 2024 guideline addresses efficacy evaluation in cattle, sheep, and goats [97,102]. These guidelines support appropriate efficacy assessment; they do not establish equivalent efficacy across drugs, parasites, or farms. Parasite removal may also precede recovery of damaged tissues and production performance [32,60]. Sustainable control therefore requires diagnosis, efficacy monitoring, responsible treatment, and complementary management.

### 6.2. Enhancement of Host Resilience and Genetic Adaptation

Improving host resistance and resilience represents a complementary strategy to direct parasite control [103,104]. Sheep-specific reviews and a meta-analysis support a heritable component to resistance against gastrointestinal nematodes, while showing that relationships with performance depend on the resistance trait measured [104,105]. Selection based on fecal egg counts or other validated indicators can therefore contribute to long-term control, but increased immune responsiveness should not automatically be equated with improved productivity [105]. Studies of divergently selected sheep also illustrate potential body-condition trade-offs [106]. Genetic improvement should be regarded as an evidence-supported, long-term complementary strategy whose implementation must consider production objectives and environmental conditions, rather than as an immediate substitute for parasite-targeted control.

### 6.3. Nutritional Strategies for Restoration of Production Efficiency

Nutritional management plays a central role in mitigating the production consequences of parasitic infections [29,72,79,107]. In particular, supplementation strategies targeting amino acid balance, energy density, and micronutrient availability can support performance in some parasite-challenged sheep and goat systems [51,79,108]. Trace elements such as iron, zinc, and selenium are of particular relevance due to their involvement in immune function and antioxidant defense systems [109]. Adequate dietary protein and energy supply can partially offset the increased nutritional demands associated with immune activation and tissue repair [71,79], and targeted supplementation may support productive performance under parasite challenge. However, the magnitude of benefit depends on baseline nutritional status, infection burden, physiological stage, and diet composition. Nutritional management should therefore be regarded as an evidence-supported supportive intervention for maintaining host function and productive capacity under infection pressure, rather than as a direct antiparasitic strategy or a replacement for parasite-targeted control.

### 6.4. Emerging Microbiota-Targeted Interventions and Metabolic Modulation

Studies in sheep and goats associate gastrointestinal parasitic infection with changes in microbial communities, nutrient utilization, and metabolite profiles [21,47,48,49]. These associations have motivated investigation of dietary and microbiota-targeted approaches. Experimental lamb studies of fermented or plant-derived feeds report changes in parasite-related outcomes, microbiota, and metabolites [14,15,16,110], but such effects may reflect multiple actions of the intervention and do not establish the microbiota as the causal therapeutic target.

However, the evidence supporting these approaches as practical interventions for parasitic disease in sheep and goats remains substantially less developed than that supporting established parasite-control measures. Studies of probiotic strains such as *Clostridium butyricum* demonstrate the potential to modulate intestinal microbial communities, nutrient utilization, and host physiological responses in goats [111,112], but such findings do not by themselves establish parasite-specific therapeutic or production benefits. Controlled studies directly evaluating parasite burden, disease severity, production outcomes, reproducibility, and field effectiveness remain limited. Microbiota-targeted interventions should therefore currently be regarded as promising experimental or supportive approaches rather than established parasite-management tools. Further parasite-specific challenge studies and field validation are required before their routine application can be recommended.

### 6.5. Integrated Parasite Management and Emerging Precision Livestock Technologies

Integrated parasite management combines established measures such as strategic or targeted treatment, pasture and environmental management, and parasite surveillance to reduce infection pressure while limiting unnecessary drug use and slowing the development of anthelmintic resistance. These approaches have direct practical relevance to current small-ruminant production systems, with efficacy monitoring guided by standardized methods and treatment decisions evaluated in their production context [57,97,102].

By contrast, technology-enabled precision livestock management represents a developing extension of conventional parasite surveillance. Sensor-based monitoring, automated assessment of animal performance, digital decision-support systems, and integration of parasitological and production data may improve the identification of animals at risk and support more targeted interventions [113,114]. However, much of the available evidence concerns precision livestock farming and animal monitoring more broadly rather than validated parasite-specific applications in sheep and goats. Evidence that these technologies consistently improve parasite-control outcomes, production efficiency, or cost-effectiveness under commercial field conditions remains limited. Precision technologies should therefore be viewed as promising enabling tools that may complement established diagnostic and management practices rather than as established parasite-control interventions.

### 6.6. From Parasite Elimination to Biological Efficiency Optimization

Taken together, an evidence-informed management perspective may extend the objective of parasite control beyond reduction in parasite burden to include maintenance of host physiological function and biological efficiency [100]. In this context, biological efficiency provides a host-centered framework for considering how parasite control, nutritional support, and other complementary interventions may influence nutrient utilization, maintenance requirements, resource allocation, and ultimately productive performance. This perspective recognizes that, in some parasite–host systems, measurable production effects can occur in the absence of overt clinical disease, although their magnitude depends on infection burden, host susceptibility, and production conditions [10,31,115]. Importantly, these intervention categories should not be regarded as equivalent in their evidence maturity or readiness for routine implementation. Established parasite-targeted and integrated control measures should remain the foundation of parasite management, while nutritional support and genetic improvement can provide evidence-supported complementary benefits. Microbiota-targeted interventions and technology-enabled precision management represent promising extensions of this framework but currently require greater parasite-specific and field-level validation. Thus, optimization of biological efficiency does not imply replacing established parasite control, but rather provides a framework for integrating interventions according to the strength of supporting evidence, practical feasibility, and production context.

## 7. Conclusions and Future Perspectives

Depending on the parasite–host system and infection context, chronic or subclinical parasitic infections can constrain host biological efficiency through impaired nutrient acquisition or utilization, increased physiological maintenance and tissue-repair demands, and altered allocation of resources between host defense and productive functions. These host-level constraints may be associated with poorer feed efficiency, slower growth, impaired reproductive performance, and reduced product yield or quality and, when sufficiently severe or persistent, may contribute to cumulative production losses at the flock level. However, the magnitude of these effects varies substantially among parasite–host combinations and production settings, and their broader economic consequences have been directly quantified for only a subset of parasitic infections. Distinguishing host-level impairment of biological efficiency from its downstream production and economic consequences is therefore important when interpreting the overall burden of parasitic infection.

This review provides an integrative perspective linking parasite-specific pathogenic processes, host physiological impairment, and production losses in sheep and goats. Although parasitic infections differ in their life cycles, tissue tropisms, and pathological effects, some parasite–host systems show overlapping downstream responses, including oxidative imbalance, gut microbiota alterations, and metabolic changes. The strength and directness of evidence for these responses vary, and their causal contributions to production losses remain incompletely established, particularly for microbiota-associated and metabolic pathways. These observations may inform the development of complementary approaches targeting host function and host–parasite–microbiota interactions, but their practical value requires parasite-specific experimental and field validation. Established parasite-control measures should remain the foundation of management.

Several critical knowledge gaps remain before the hidden economic burden of parasitic infections can be accurately quantified and effectively mitigated. First, field-based assessment of chronic and subclinical production losses remains insufficient, particularly across diverse environmental and management systems. Future studies should evaluate whether longitudinal epidemiological surveillance combined with precision livestock technologies can improve the detection and management of parasite-associated production losses across diverse field conditions. Second, the mechanistic links between parasite-induced microbial disruption, microbial metabolites, and systemic host outcomes require further experimental validation. Integrating multi-omics approaches, including metagenomics, metabolomics, and host transcriptomics, may help identify candidate biomarkers and putative intervention targets relevant to reduced biological efficiency. However, their reproducibility, causal relevance, and utility for predicting or improving production outcomes in sheep and goats require independent validation before practical application. Third, translating molecular insights into practical production strategies requires stronger integration between mechanistic parasitology, animal nutrition, breeding programs, and farm-level management.

Future parasite research should therefore advance toward production-oriented systems parasitology within a One Health framework, where parasitic infections are considered not only as animal health problems but also as challenges affecting food security, agricultural sustainability, and resilient livestock production. Future control frameworks should retain validated parasite-control and management measures as their foundation while determining where nutritional and genetic strategies provide additional benefit and whether emerging microbiota- and precision-based approaches can offer reproducible advantages under field conditions. For the latter approaches, parasite-specific efficacy, scalability, cost-effectiveness, and practical implementation require further evaluation before routine recommendation.

## Figures and Tables

**Table 1:** Major parasitic groups affecting sheep and goats and their integrated impacts on host physiology and production efficiency.

Parasite Group	Representative Parasites	Primary Target System	Major Physiological Alterations	Production Consequences	Ref.
Gastrointestinal parasites	*Eimeria* spp.; *Haemonchus contortus*; *Ostertagia* spp.; *Trichostrongylus* spp.; *Moniezia* spp.	Intestinal epithelium and gastrointestinal tract	Epithelial injury, blood and protein loss, impaired nutrient utilization; relative importance varies by parasite	Reduced feed intake, decreased feed conversion efficiency, growth retardation, lower production performance	[21,28,30,32,33,34]
Hepatobiliary and visceral parasites	*Fasciola hepatica*; *Echinococcus granulosus*	Liver and biliary system (*F. hepatica*); liver, lungs, and other visceral organs (*E. granulosus*)	Hepatic or biliary injury; cyst-associated tissue compression and organ condemnation	Poor condition or growth in fluke disease; organ condemnation and reduced slaughter value	[25,35,36,37,38]
Blood and tissue parasites	*Babesia* spp.; *Theileria* spp.; *Toxoplasma gondii*; *Sarcocystis* spp.	Blood, placenta, skeletal muscle and systemic tissues	Hemolysis, anemia, immune dysregulation, placental damage, muscle fiber degeneration	Anemia and impaired condition; reproductive loss in toxoplasmosis; muscle lesions and altered flavor-associated metabolites in sarcocystosis	[23,26,39,40,41,42]
Ectoparasites	Ticks; mites; lice; myiasis-causing flies	Skin, wool and peripheral tissues	Blood loss, dermatitis, skin lesions, persistent irritation, secondary bacterial infection	Skin and product damage, impaired welfare, and additional management needs	[11,27,43,44]
Potential overlapping host responses	Multiple parasite groups	Multi-organ systems	Chronic immune activation, oxidative imbalance, impaired nutrient utilization, and context-dependent metabolic alterations	Possible contributions to impaired performance; direct causal and economic evidence varies	[21,22,45,46,47,48,49]

**Table 2:** Hierarchical framework linking impaired host biological efficiency to production and economic consequences of parasitic infections in sheep and goats.

Level	Production Domain	Representative Manifestations	Biological Basis	Economic Implication	Ref.
Individual level	Growth performance	Reduced ADG, increased FCR, delayed market weight	Impaired nutrient absorption, chronic inflammation, increased maintenance energy expenditure	Reduced feed efficiency and lower production value	[21,30,51]
	Reproductive performance	Reduced conception rate, abortion, decreased lamb survival	Endocrine disruption, energy redistribution, placental or systemic infection	Reduced reproductive output and flock replacement efficiency	[39,42,57]
	Product quality	Altered meat quality in selected studies; reduced wool production and milk yield	Parasite-specific tissue injury or physiological impairment; experimental co-exposure must be considered	Lower carcass value and product marketability	[27,30,41,58,59]
Population level	Herd stability	Increased mortality, culling, and herd instability	Severe disease in susceptible animals, including anemia in haemonchosis	Increased replacement costs and reduced long-term productivity	[11,32]
System level	Hidden production losses	Subclinical infection, reduced feed efficiency, prolonged production cycle	Persistent metabolic inefficiency without obvious clinical signs	Underestimated cumulative production losses	[30,31]
	Management and control cost	Anthelmintic treatment expenditure	Ongoing control of helminth infections	Increased production costs and reduced profitability	[7]
	Resistance	Reduced anthelmintic efficacy	Selection pressure under repeated drug use	Higher control costs and sustainability challenges	[7,17,18,19]

**Table 3:** Selected study-specific observations of intestinal alterations associated with *Eimeria* spp. infection in sheep, distinguishing experimental challenge models from naturally occurring infection.

Host	*Eimeria* spp.	Infection Context/Exposure	Intestinal Site Assessed	Study-Specific Observations	Ref.
3–4-week-old coccidia-free lambs	*E*. *crandallis*	Experimental challenge and diclazuril-treatment study; multiple doses of sporulated oocysts	Small intestine and cecum	In untreated infected lambs, first-generation meronts were associated with loss of surface epithelial cells and villous atrophy, whereas pro-gamont stages were associated with crypt destruction and crypt hyperplasia.	[65]
19–21-day-old lambs	*E*. *ovinoidalis*	Experimental challenge in two experiments: 2.5 × 10^5^ or 1 × 10^5^	Ileum, particularly ileal Peyer’s patches	Ileal Peyer’s patch follicles were largely normal approximately 2 weeks after infection. At approximately 3 weeks, follicle-associated epithelium showed focal hyperplasia and occasional necrosis; lymphocyte depletion occurred in ileal lymphoid follicles, with reduced follicle size in some lambs.	[66]
50-day-old female lambs	*E*. *ovinoidalis*	Experimental 2 × 2 factorial challenge with *E*. *ovinoidalis* and AFB1; 2 × 10^4^ sporulated oocysts, with or without AFB1 exposure	Ileum, cecum and colon; barrier, immune and microbiota analyses focused mainly on the cecum	The experimental model demonstrated intestinal epithelial and cecal barrier injury in challenged lambs. AFB1 co-exposure further aggravated several coccidia-associated changes, including goblet-cell and tight-junction alterations, inflammatory responses, microbiota disruption, and reduced SCFA production. These findings therefore reflect a co-exposure model and should not be attributed uniformly to *E*. *ovinoidalis* alone.	[22]
Lamb from an affected sheep flock	*E*. *granulosa*	Naturally occurring outbreak/case investigation; no experimental inoculation dose	Duodenum, jejunum and ileum	Necropsy revealed scattered petechiae and white spots visible through the serosal surface of the small intestine. *E*. *granulosa* was identified by parasitological and molecular methods. Affected lambs in the flock showed clinical signs including diarrhea, anorexia, weight loss, and weakness/paralysis.	[67]
35-day-old female Hu lambs	*E*. *ovinoidalis*	Experimental dose–response challenge: 1 × 10^4^, 3 × 10^4^, or 1 × 10^5^ sporulated oocysts; uninfected control	Ileum, cecum and colon; microbiota and metabolomic analyses primarily compared high-dose and control groups	Clinical severity generally increased with challenge dose. In high-dose lambs, sequential parasite development was observed in the ileum, cecum and colon, with marked epithelial loss, villous disruption, crypt damage, and lamina propria hemorrhage/congestion at peak intestinal pathology. The high-dose group also showed time-dependent changes in serum cytokines, fecal microbiota, and metabolomic profiles relative to controls.	[21]

Note: These are selected study-specific observations in sheep. Findings depend on parasite species or strain, challenge dose, host age, sampling time, infection context, and concurrent exposures. Controlled challenges, co-exposure experiments, and naturally occurring infections should not be interpreted as equivalent or generalized to all Eimeria infections.

**Table 4:** Major biological mechanisms linking parasitic infections to reduced production efficiency in sheep and goats.

Mechanistic Category	Representative Parasites	Primary Biological Alterations	Consequences for Production Efficiency	Ref.
Intestinal epithelial destruction	*Eimeria* spp.	Villus atrophy, epithelial desquamation, tight junction disruption, increased intestinal permeability, mucus layer impairment	Reduced nutrient digestion and absorption, decreased feed efficiency, growth retardation	[21,22,33,50,65,66]
Blood loss and anemia	*Haemonchus contortus*	Chronic blood loss, anemia, hypoproteinemia, reduced oxygen transport, protein depletion	Reduced growth, impaired reproduction, increased mortality and management costs	[32,45]
Hepatobiliary and visceral organ dysfunction	*Fasciola hepatica*, *F. gigantica*, *Dicrocoelium dendriticum*, *Echinococcus granulosus*	Hepatic injury and fibrosis in fasciolosis; biliary involvement without parenchymal migration in dicrocoeliosis; cyst-associated compression in echinococcosis	Reduced feed efficiency, organ condemnation, lower carcass value, chronic productivity decline	[35,36,37,38]
Impaired nutrient availability and utilization	*Moniezia expansa*, *Trichostrongylus* spp., *Teladorsagia circumcincta*, *Nematodirus* spp.	Altered digestion, absorption and feeding behavior; possible nutrient competition by cestodes	Reduced performance documented more directly for nematodes; cestode-related production effects less firmly quantified	[28,34,52,71,72,73]
Immune–metabolic trade-offs	*Babesia* spp., *Theileria* spp.	Anemia, inflammation, fever and anorexia; possible resource-allocation costs	Impaired condition and performance; direct quantification of immune–metabolic costs remains limited	[11,23,26,71,74,75]
Potentially overlapping downstream host responses	Multiple parasite groups	Oxidative imbalance, context-dependent gut microbiota alterations, altered SCFA profiles, and host metabolic changes	Possible contributions to impaired nutrient utilization and performance; causal evidence varies	[21,22,45,46,47,48,49,76,77]

## Data Availability

No new data were created or analyzed in this study. Data sharing is not applicable to this article.

## Abbreviations

ADG	Average daily gain
FCR	Feed conversion ratio
ZO-1	Zonula occludens-1
SCFAs	Short-chain fatty acids
